# Supplementary material for: Material category of visual objects computed from specular image structure
Source: Nat Hum Behav. 2023 Jun 29;7(7):1152–69. doi: 10.1038/s41562-023-01601-0 (PMC10365995; doi:10.1038/s41562-023-01601-0)
Supplement: Supplementary file 1 — Supplementary Analyses A and B, Figs. 1–19 and Tables 1–3. [file 41562_2023_1601_MOESM1_ESM.pdf]

nature human behaviour

Article

https://doi.org/10.1038/s41562-023-01601-0

# Material category of visual objects computed from specular image structure

In the format provided by the authors and unedited

# SUPPLEMENTAL ANALYSES

**Supplementary Analysis A: Regression model predicting material score from gloss level**

Further supporting the idea that material class may not be determined by but may constrain gloss, we found that a model predicting material score from gloss level performed worse than a model predicting material score directly from a linear combination of the cues for gloss (i.e., the visual features coverage, sharpness, and contrast) for most material classes (Supplementary Figure 12A (i)). The addition of colour cues led to an overall improvement in predicting material scores (Supplementary Figure 12 A (ii) and (iii)). One potential mechanism for this is that visual features independently determine gloss (gloss cues) and material (gloss cues + colour cues). However, Supplementary Figure 12E (iv) shows that, in addition to gloss cues, colour cues also contribute to perceived gloss for some materials, suggesting that cues for gloss and material might not be independent. In fact, the contribution of visual features to perceived gloss was not stable across different material classes (black bars in Figure 5C), as would be expected if the visual system computed surface gloss linearly from visual cues independent of material class. Instead, the predictiveness of each cue to gloss differed with material (Figure 5C, middle), and this covaried with how well each cue predicted material score (that is, visual features that predicted gloss also tended to predict material score; Supplementary Figure 12B (ii) and (iii)). This dependency between cues for gloss and cues for material is unlikely to be due to gloss mediating cues to material (*feedforward hypothesis*) as our previous analyses showed that gloss level was not a good indicator of material class. Nor can the differing contributions of cues to gloss be explained by the relative availability of those cues within each class (i.e., visual feature variability; Supplementary Figure 12B (i)). This implies that, rather than gloss mediating cues to material, the visual features used to estimate gloss could instead be mediated by perceived category.

**Supplementary Analysis B: RSA showing that visual features predict material changes better than gloss perception itself**

If the same features of specular image structure underlie material discrimination and gloss perception (*simultaneous hypothesis*; Figure 1B (ii)), then perceived differences in both material (Experiment 2) and gloss (Experiment 3) should be reflected by differences in the measured visual features (Figure 2). Furthermore, differences in material should not be better predicted by perceived gloss (*feedforward hypothesis*) than by visual cues for gloss directly. To test these predictions, we calculated dissimilarity scores for each pair of stimuli in terms of perceived material, perceived gloss, and measured features (i.e., each gloss cue and colour cue; Supplementary Figure 15A). Material dissimilarity scores were calculated as one minus the spearman correlation coefficient for each pair of category profiles. Gloss dissimilarity scores were calculated as the absolute difference between average gloss ratings between each stimulus pair, and dissimilarity scores for each visual feature (cue) were calculated in the same way (using absolute difference). Each point in the matrices (known as representational dissimilarity matrices or RDMs) shows how dissimilar each stimulus is to each

other stimulus in terms of their category profile, gloss ratings, and features, with yellow pairs being more dissimilar.

Dissimilarity scores in the upper triangles of each RDM (highlighted in red) were vectorized and subjected to two linear regressions that predicted material dissimilarity and gloss dissimilarity (separately) from visual feature dissimilarities (Supplementary Figure 15B). Supplementary Figure 15C shows that visual feature dissimilarities significantly predicted differences in both perceived material, $R^2$=0.32, F(6,426419)=32797, p<0.001, and perceived gloss, $R^2$=0.42, F(6,426419)=50635, p<0.001. These feature models performed slightly better than models using differences in reflectance parameters as predictors (Supplementary Figure 15C), demonstrating that the features capture the variance explainable (by a linear model) in material and gloss caused by changes in surface reflectance. The slight improvement in predictive value for the measured features over reflectance parameters is likely due to the fact that the features additionally account for differences in image structure caused by shape and lighting differences, which can affect material appearance (e.g., Norman et al., 2020). Furthermore, we found that the model containing (all six) visual features ($R^2$=0.32) predicted changes in material better than a model containing only colour cues (lowlight value, lowlight saturation, highlight saturation) in conjunction with perceived gloss, $R^2$=0.23 (Supplementary Figure 15), providing further evidence that material recognition occurs directly from image structure that (simultaneously) triggers our perception of mid-level properties like gloss, rather than via estimation of those properties in a feedforward manner.

# SUPPLEMENTAL FIGURES

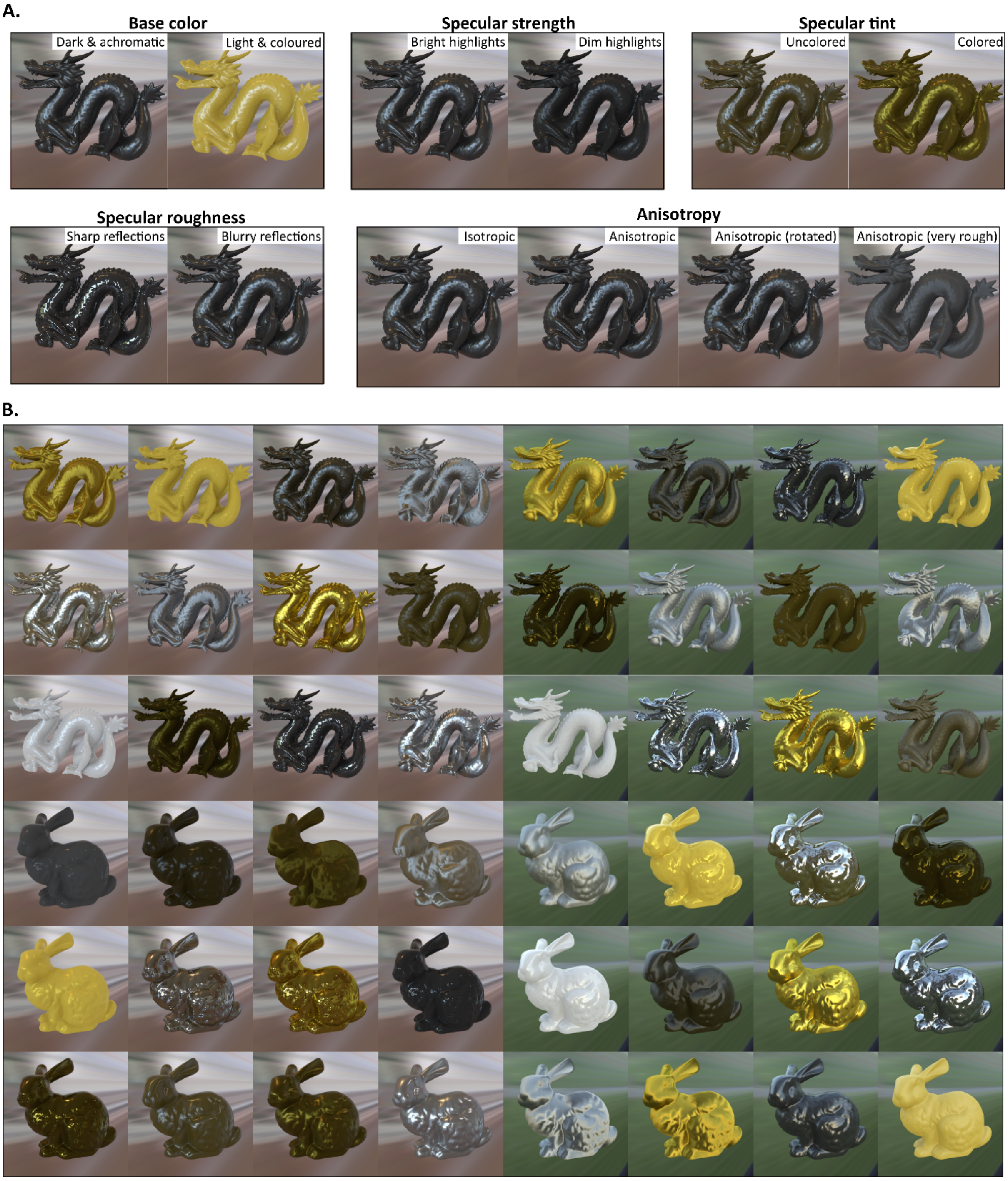


**Supplementary Figure 1.** Computer-rendered stimuli in the experiments were complex 3D shapes embedded in natural illumination fields. **A.** The visual effects of manipulating different rendering parameters. **B.** A sample of the stimuli used in the 18-AFC and gloss rating experiments (Experiments 2 and 3). Only reflectance parameters were manipulated and yet we found that these variations yielded many different perceived material classes beyond those defined by the reflectance function used.

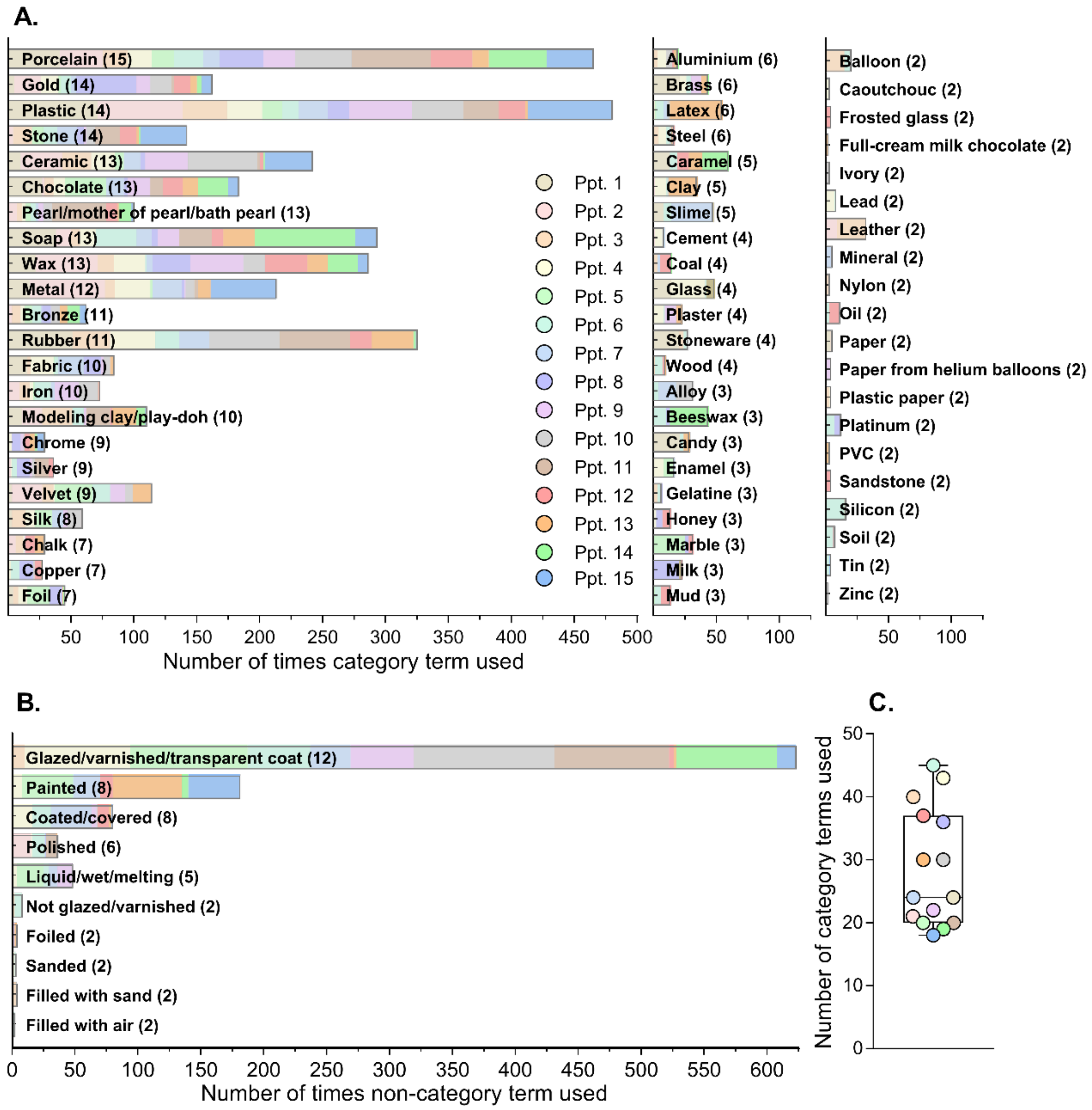


**Supplementary Figure 2.** Results of the free-naming experiment (Experiment 1, n=15). After processing for duplicates and similar terminology, 209 terms were used to describe the materials, 121 of which were *category terms* (i.e., nouns like porcelain, gold, plastic). There were 17 *non-category terms* that included descriptions of fillings (e.g., filled with sand), coatings (e.g., glazed, varnished, painted, covered), finishes (e.g., polished, sanded), and states (e.g., liquid, wet, melting). Participants also used a total of 71 *other adjectives* to describe the stimuli (e.g., dark, yellow, glossy, matte). These other adjectives were excluded from analyses altogether but can be found in Supplementary Figure 3. The bar plots show the number of times each term was used by each participant, for the 64 category terms **(A)** and 10 non-category terms **(B)** that were used by at least two participants. The numbers in brackets correspond to the number of participants (out of 15) that used each term. Not shown are the 57 category terms and 7 non-category terms that were used by only one participant, and the 71 *other adjectives*. Importantly, the use of each term was distributed quite well among participants, i.e., category labels did not come from the same few participants. The box plot in **(C)** supports this by showing that each participant used many category terms (range = 18-45, median = 24).

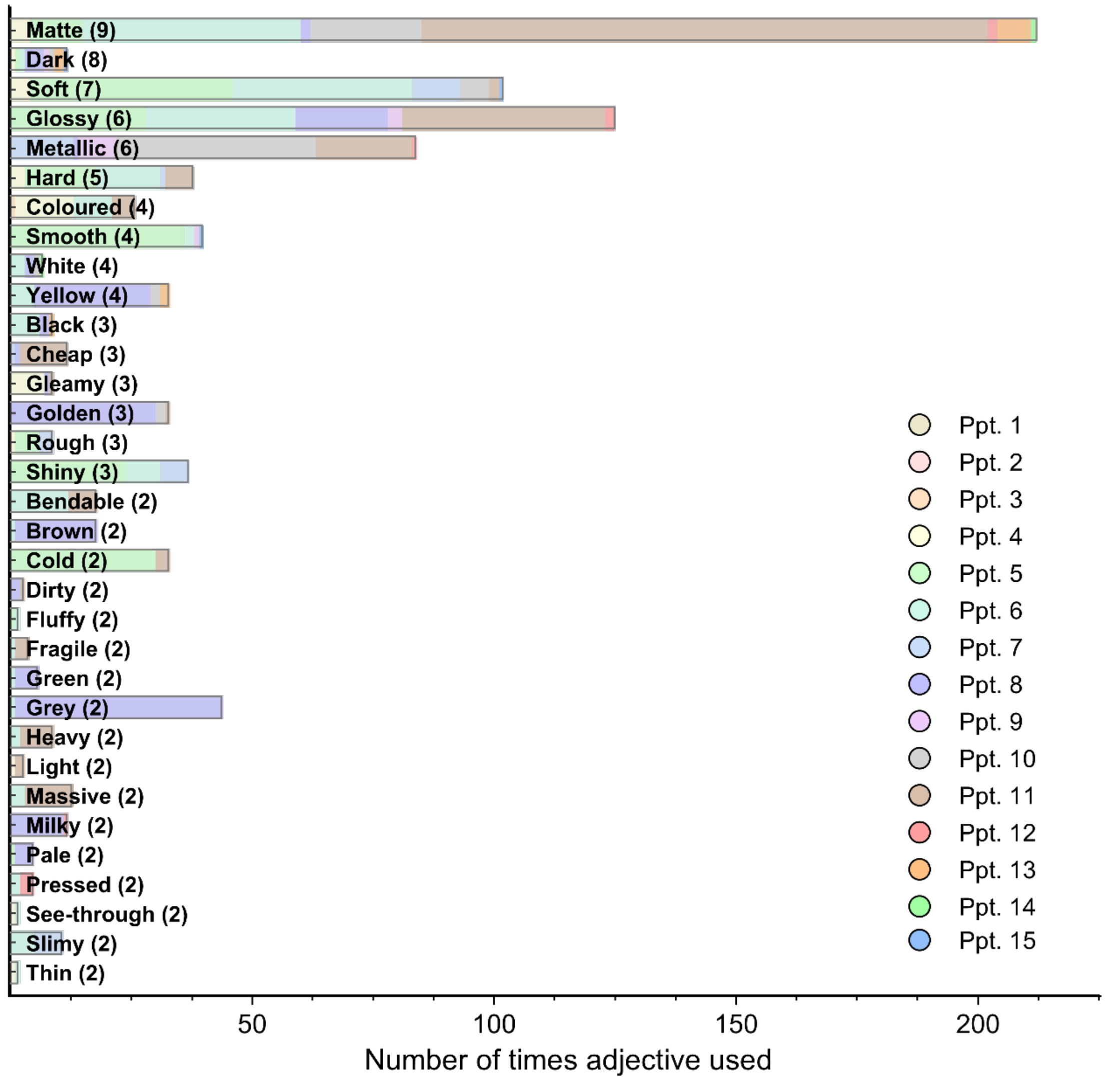


**Supplementary Figure 3.** Additional results from the free-naming experiment (Experiment 1), showing the number of times each *other adjective* term was used. Only adjectives that were used by at least two participants are shown.

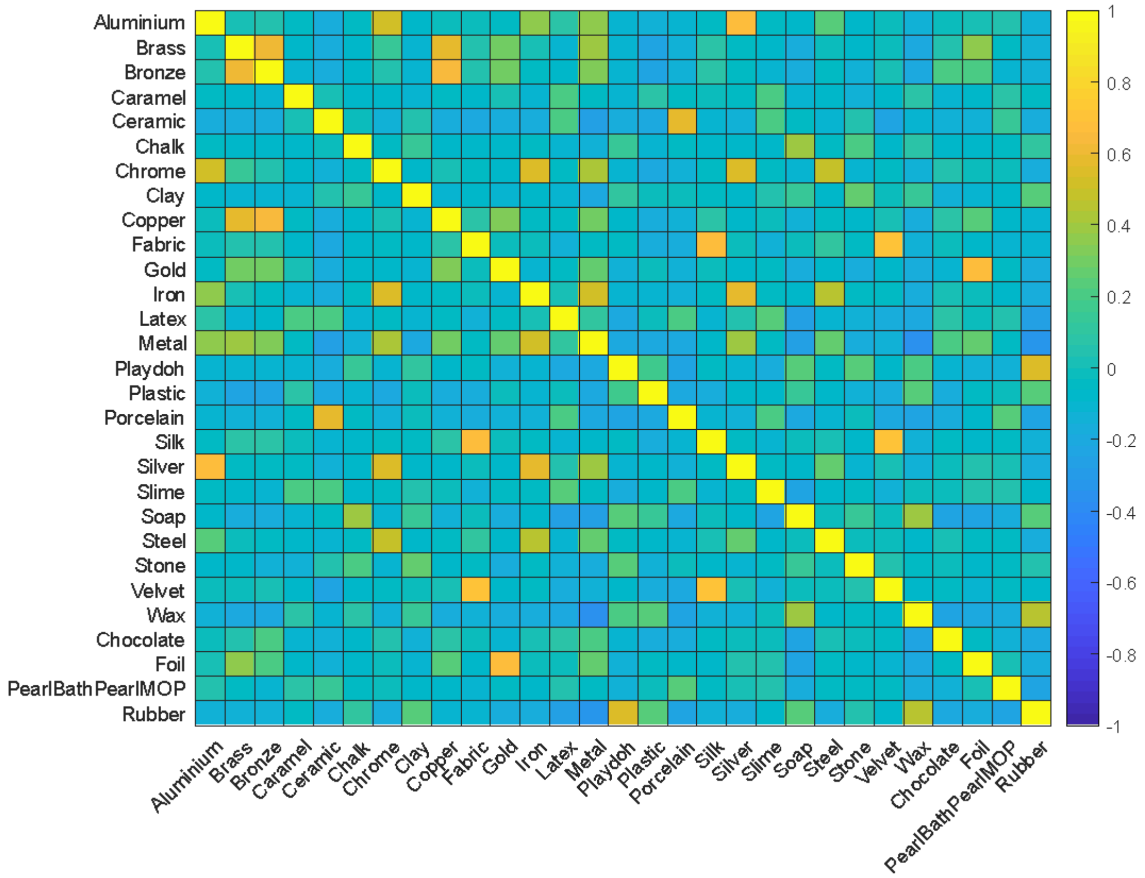


**Supplementary Figure 4.** Heat plot showing Pearson correlations between pairs of categories from the free-naming task (Experiment 1), calculated from the number of participants that used each term for each stimulus. That is, for each pair of categories, correlated vectors were indexed by stimulus number with the values being number of observer responses. These correlations were used to guide the merging of visually/semantically similar category terms for the 18-AFC task (Experiment 2).

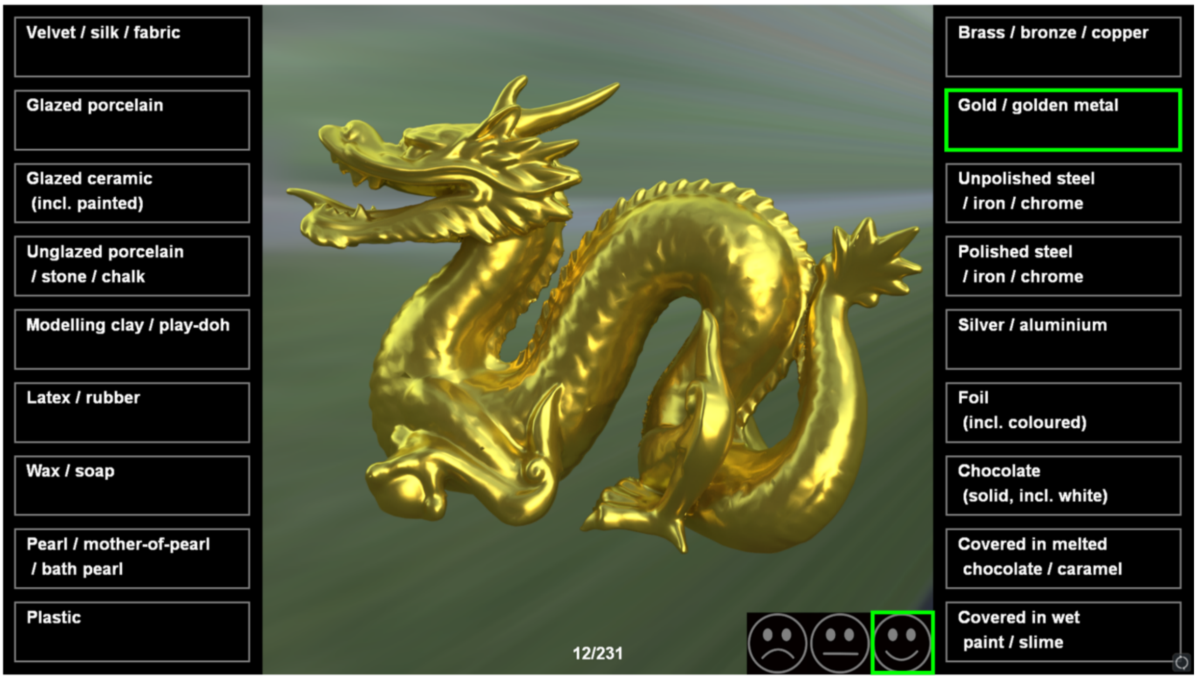


**Supplementary Figure 5.** A screenshot of an example trial from the 18-AFC experiment (Experiment 2). Observers were presented one stimulus at a time and were asked to choose the category that best applied to each stimulus. If they were not perfectly happy with their category choice (e.g., if the category they were looking for was not an option), they could adjust their confidence rating at the bottom right of the screen accordingly. At the bottom centre of the screen was a trial counter indicating the current trial and total number of trials in that block. Note that the categories were presented in German in the actual experiment. The German translations are as follows, in the order shown in the figure (1-9 displayed on the left; 10-18 displayed on the right):

1) Samt / Seide / Stoff
2) Glasiertes Porzellan
3) Glasiertes Steingut / Keramik
4) Unglasiertes Porzellan / Stein / Kreide
5) Knete / Modelliermasse
6) Latex / Gummi
7) Wachs / Seife
8) Perle / Perlmutt / Badeperlen
9) Plastik / Kunststoff
10) Messing / Bronze / Kupfer
11) Gold / goldenes Metall
12) Unpolierter(s) Stahl / Eisen / Chrom
13) Hochpolierter(s) Stahl / Eisen / Chrom
14) Silber / Aluminium
15) Folien (auch farbige)
16) Schokolade (fest, auch weisse)
17) mit flüssiger(m) Schokolade / Karamel überzogen
18) mit flüssiger(m) Farbe / Schleim bedeckt

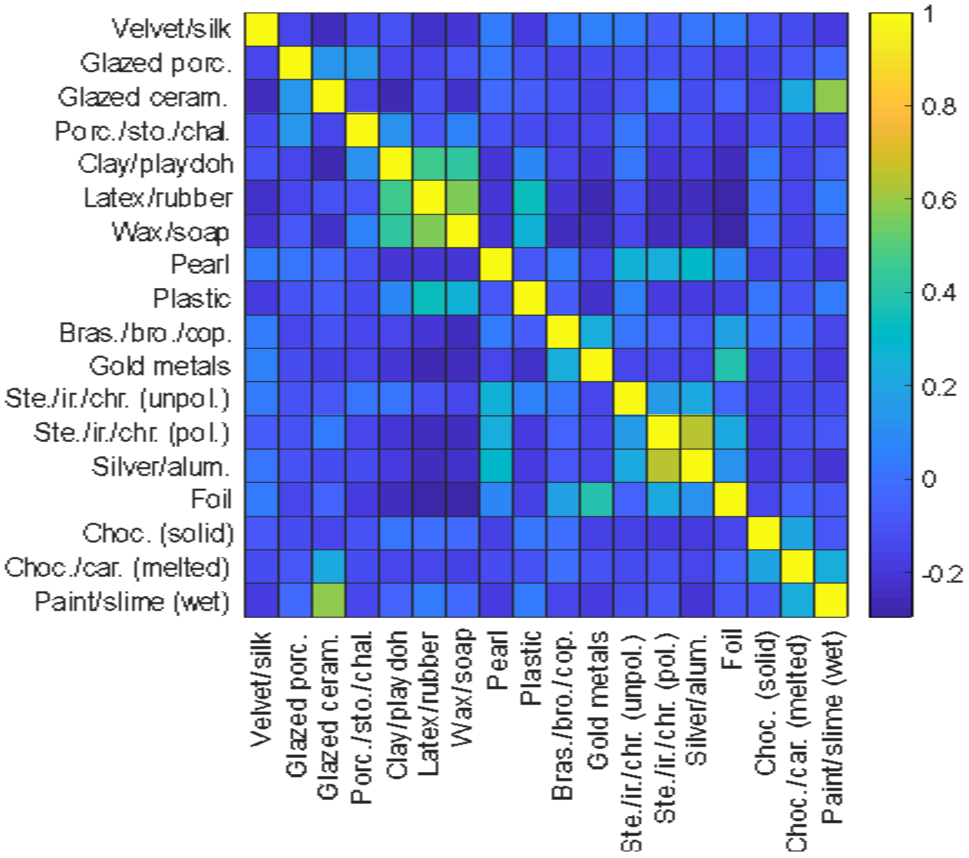


**Supplementary Figure 6.** Heat plot showing Pearson correlations between stimulus profiles (confidence rating sums) for each pair of categories from the 18-AFC experiment (Experiment 2).

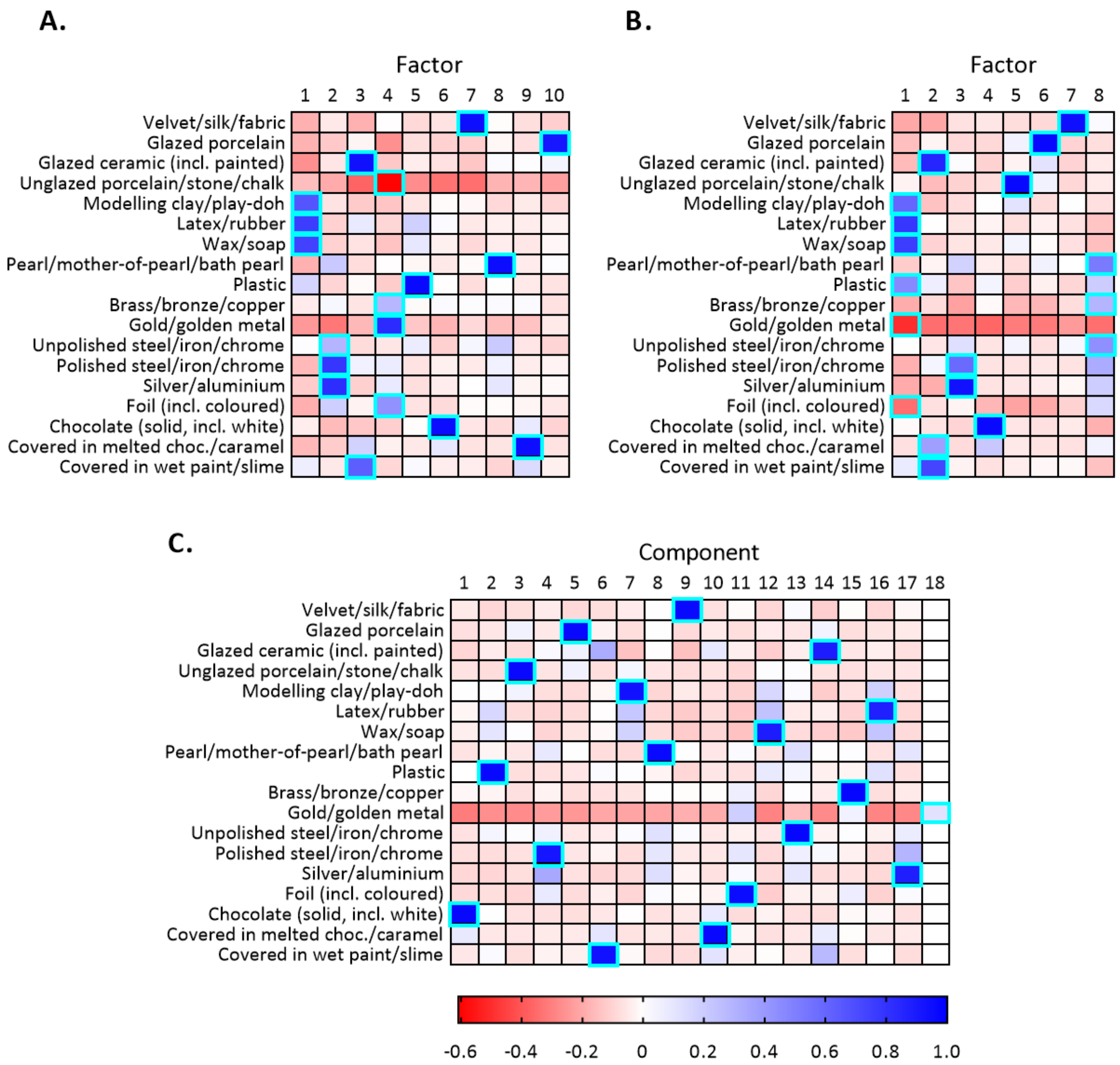


**Supplementary Figure 7.** Heat plots of loadings for each category from the 18-AFC task when different numbers of dimensions are retained. The highlighted cells show the dimension onto which each category most strongly loaded. The emergent dimensions from each analysis are comparable to the 12-factor solution in Figure 4 in terms of their interpretation. **A.** 10-factor solution: (1) Rubber-like; (2) Uncolored metals; (3) Ceramics; (4) Gold metals; (5) Plastic; (6) Solid chocolate; (7) Velvety/silky; (8) Pearlescent; (9) Melted chocolate, (10) Glazed porcelain; (11) Unglazed porcelain **B.** 8-factor solution: (1) Plastic/rubber-like; (2) Ceramics; (3) Uncolored metals; (4) Solid chocolate; (5) Unglazed porcelain; (5) Glazed porcelain; (6) Velvety/silky; (7) Pearlescent/unpolished metals. **C.** Principal components analysis solution retaining all categories.

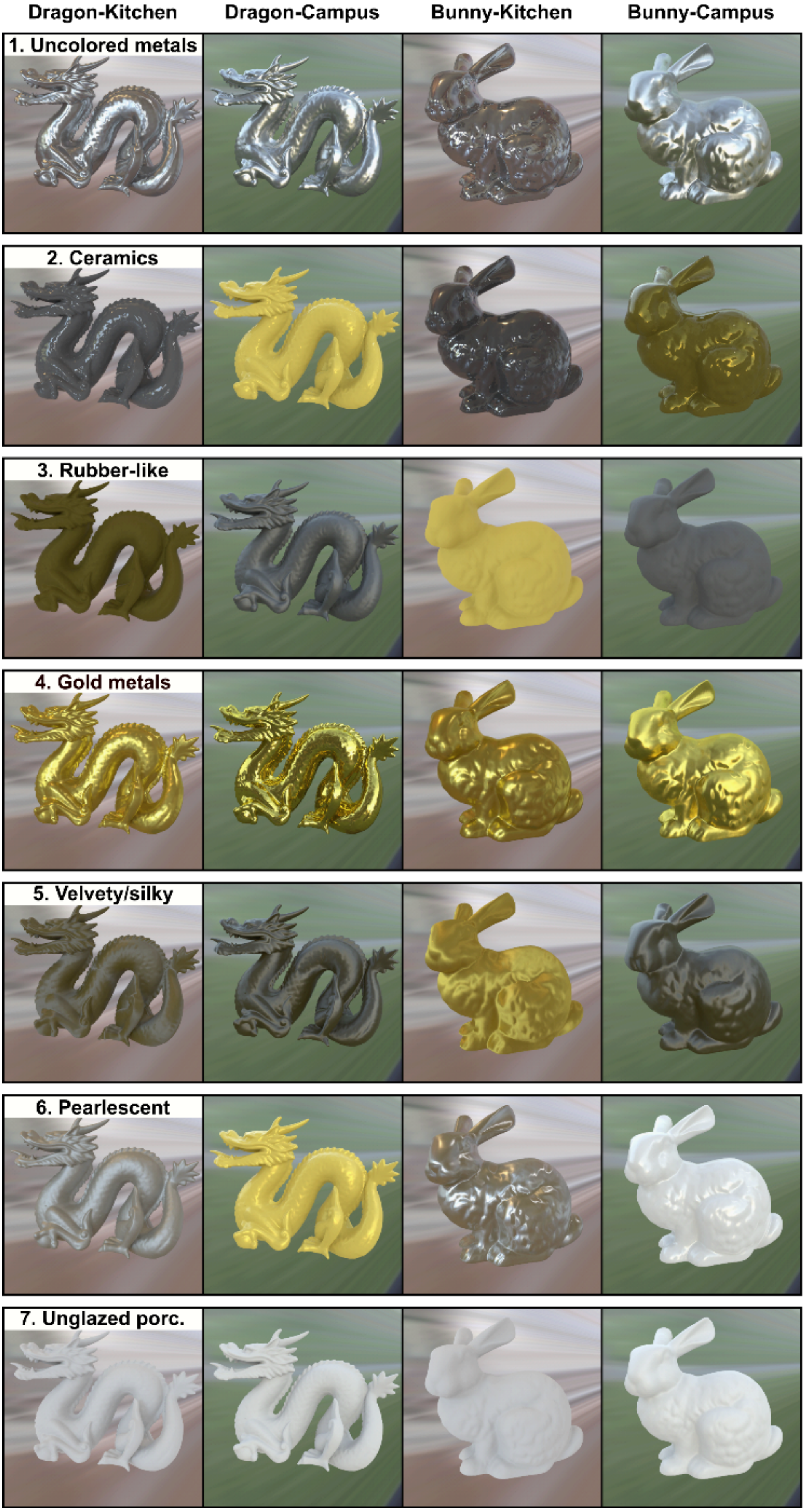
Dragon-Kitchen
Dragon-Campus
Bunny-Kitchen
Bunny-Campus
1. Uncolored metals
2. Ceramics
3. Rubber-like
4. Gold metals
5. Velvety/silky
6. Pearlescent
7. Unglazed porc.

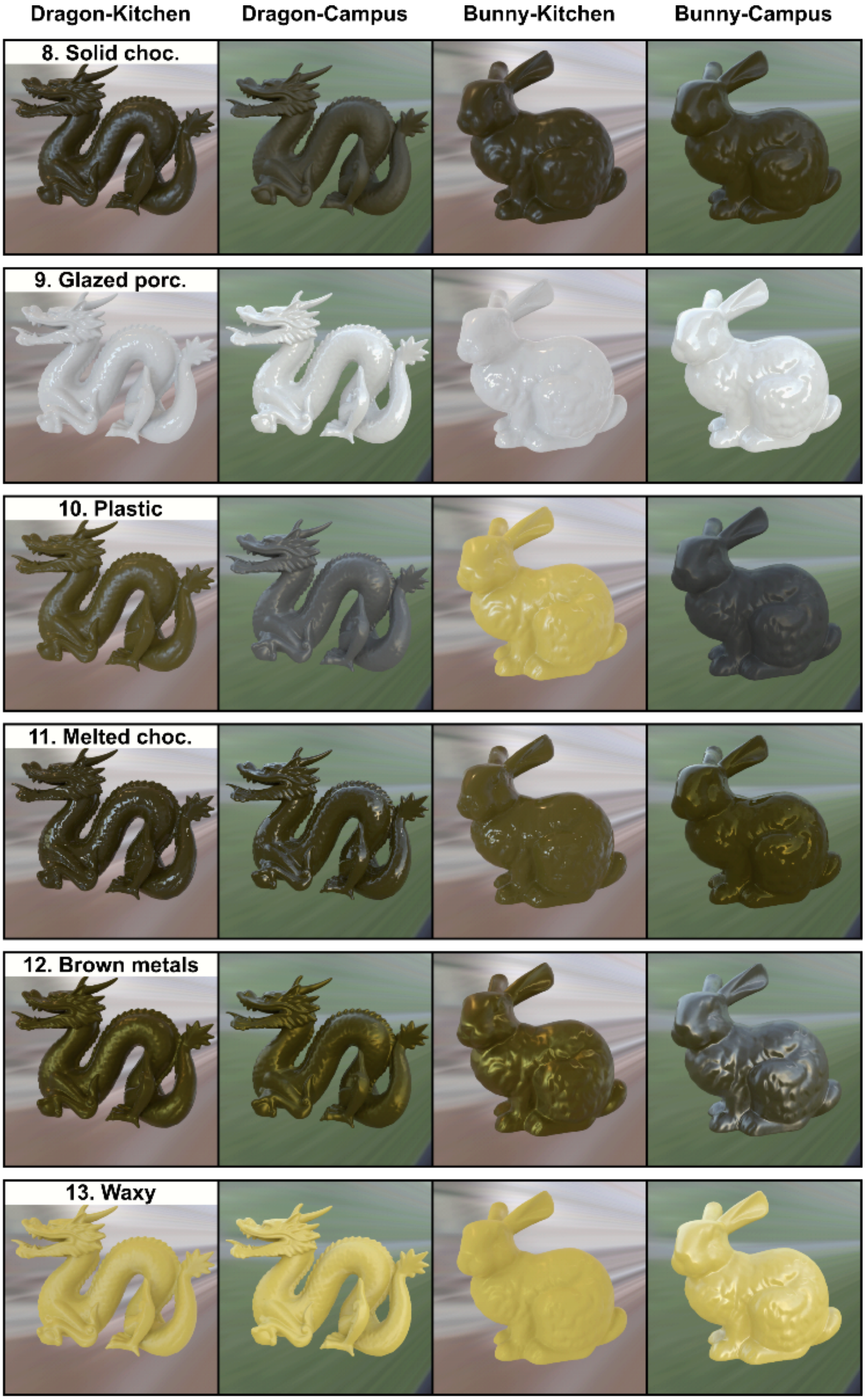


**Supplementary Figure 8.** More example stimuli from each emergent dimension from the factor analysis (Figure 4), for each shape and light field condition.

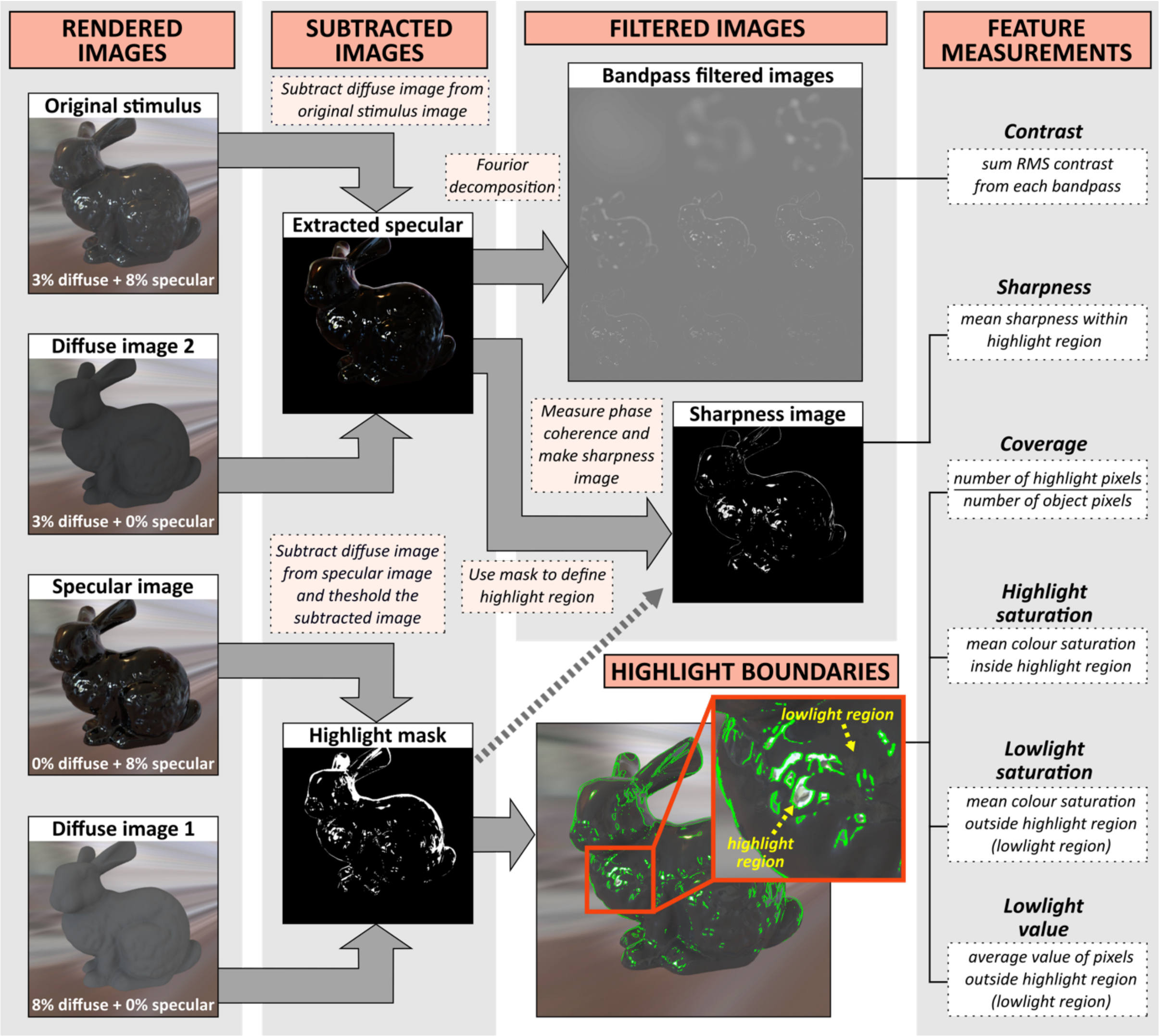


**Supplementary Figure 9.** Calculation of the six specular reflection visual features. See Analyses for details. **First column:** To help with segmenting the image structure caused by specular reflections and diffuse shading, we rendered additional images that isolated specular and diffuse components. **Second column:** We extracted both specular highlights (bright reflections; "Highlight mask" image) and specular reflections, which include both specular highlights and lowlights (i.e., both bright and dim reflections; "Extracted specular" image). **Third column:** The bottom image shows the results of the specular highlight segmentation, with the green outline showing the boundary between highlights and lowlights. The top images show filtered images that are outputted as intermediate steps in the calculation of contrast and sharpness visual features. **Last column:** *Coverage* was defined as the proportion of object pixels that were calculated to be specular highlights (excluding lowlights), based on a threshold above the maximum diffuse shading. *Contrast* was the sum of root-mean-squared (RMS) contrast of extracted specular reflections at different spatial frequency bandpasses. *Sharpness* of extracted specular reflections was calculated for each pixel within the highlight regions using a measure of local phase coherence (Hassen, Wang, & Salama, 2013), then these values were averaged. *Highlight saturation* and *lowlight saturation* were calculated as the average colour saturation of pixels within the highlight region, and outside of the highlight region (which we call the lowlight region), respectively. *Lowlight value* was calculated as the average value (or lightness) of pixels in the lowlight region.

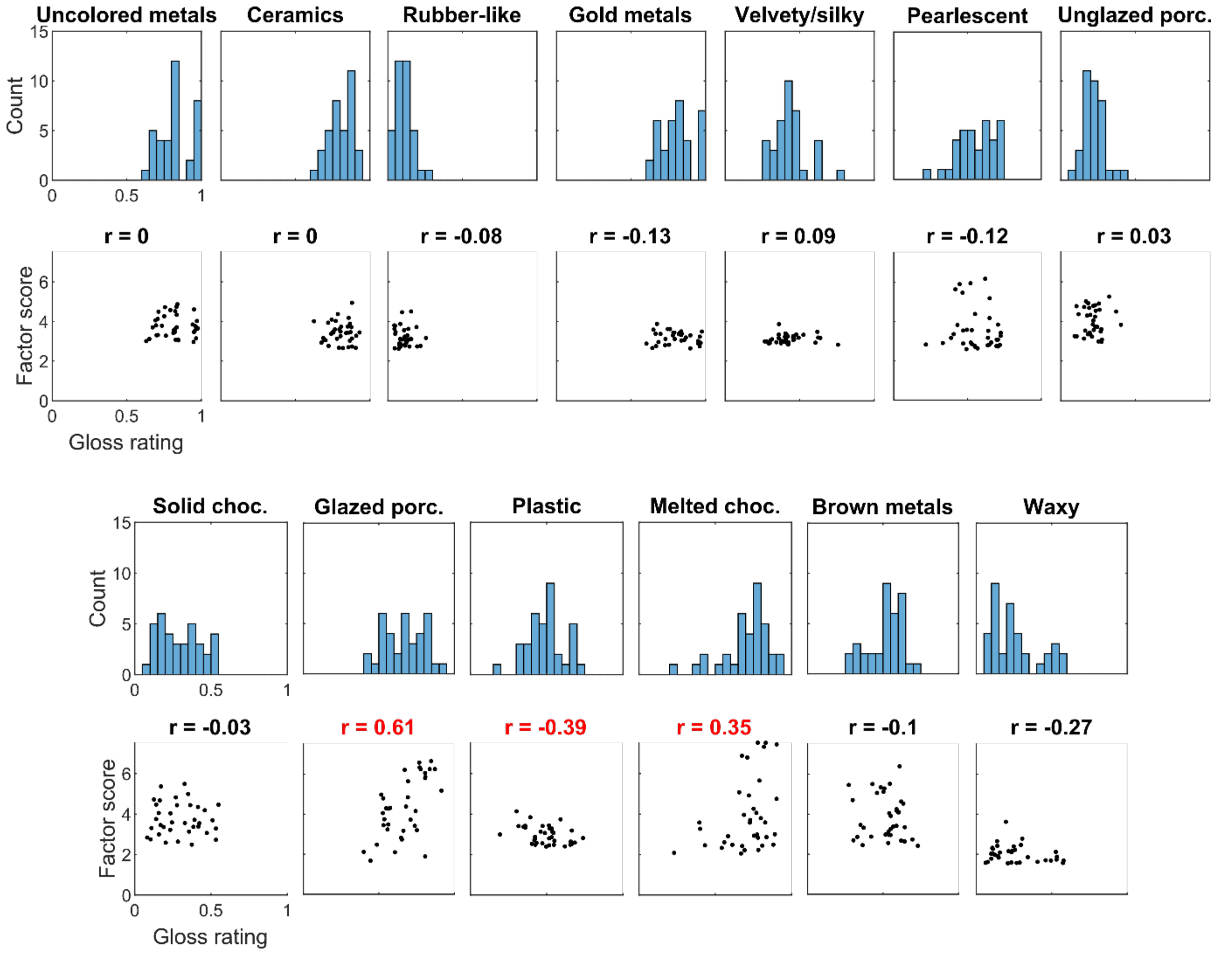


**Supplementary Figure 10.** Histograms and corresponding scatter plots show gloss ratings and correlations between gloss ratings and factor scores (12 factor solution) for the top loading stimuli (cut-off=36). For this more conservative analysis it is apparent that, overall, stimuli from the same material class exhibited a wide distribution of gloss levels, as we see it also in the more inclusive plots in Figure 5 and Supplementary Figure 11.

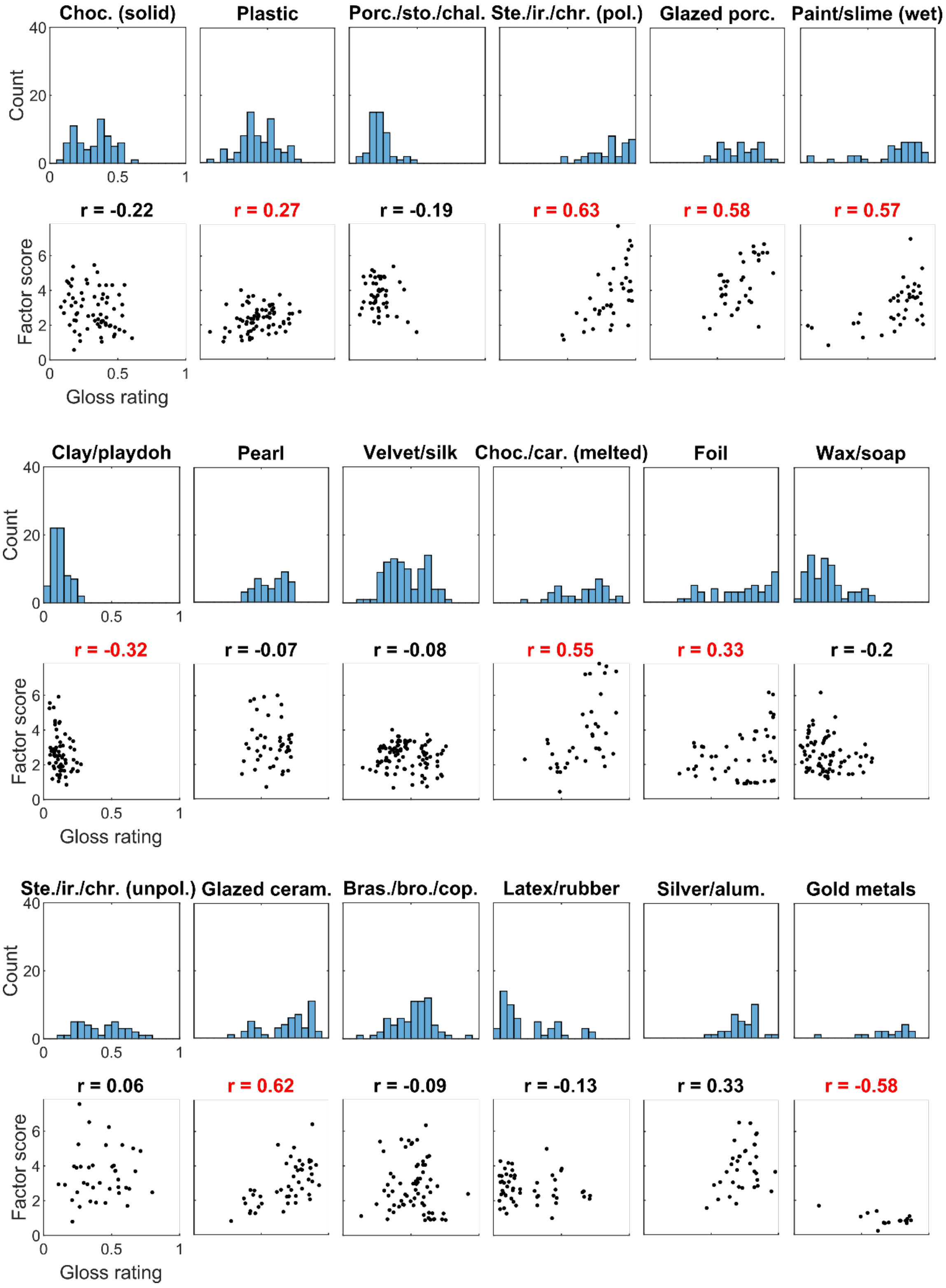


**Supplementary Figure 11.** Histograms and corresponding scatter plots show gloss ratings and correlations between gloss ratings and scores (18 component solution) for all stimuli. The general pattern that we saw in Figure 5 and Supplementary Figure 10 persists, i.e., that stimuli from the same material class exhibit a wide distribution of gloss levels. The same holds true for other factor solutions.

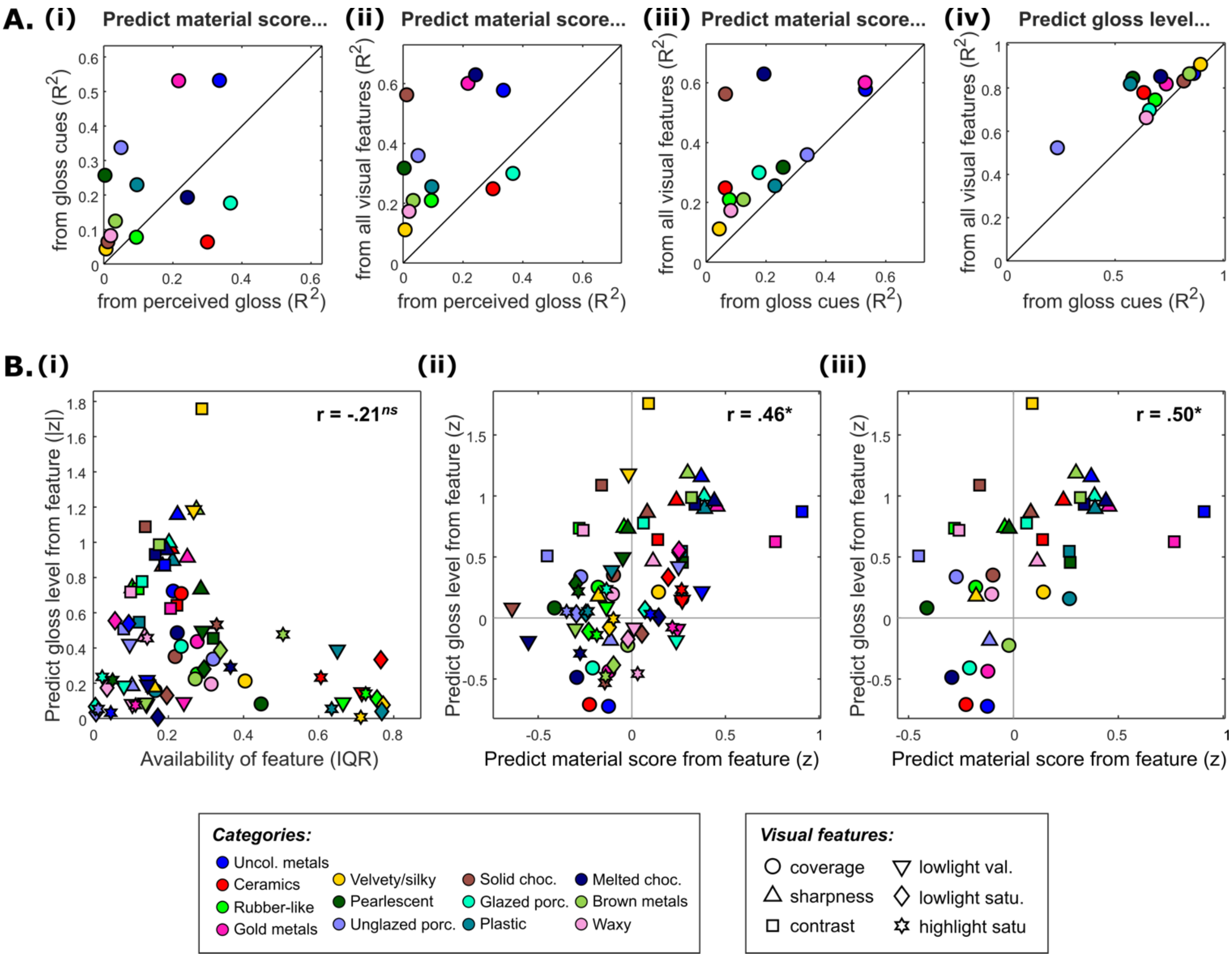


**Supplementary Figure 12. A.** Gloss cues predict material score (adjusted $R^2$) better than gloss ratings (perceived gloss) for most categories (i) and adding colour cues improves this (i.e., "all visual features"; ii and iii). Furthermore, gloss ratings are sometimes better predicted with the inclusion of colour cues (i.e., "all visual features", iv). **B.** The predictiveness of visual features for gloss ratings is unrelated to the "availability" (as measured by interquartile range; IQR) of those features within a given class (i), but *are* related to the visual features that predict material (ii – all six features; iii – three gloss cues). ' z' stands for Fisher-transformed correlation coefficients (Pearson correlation). Datapoints are colour coded by material category.

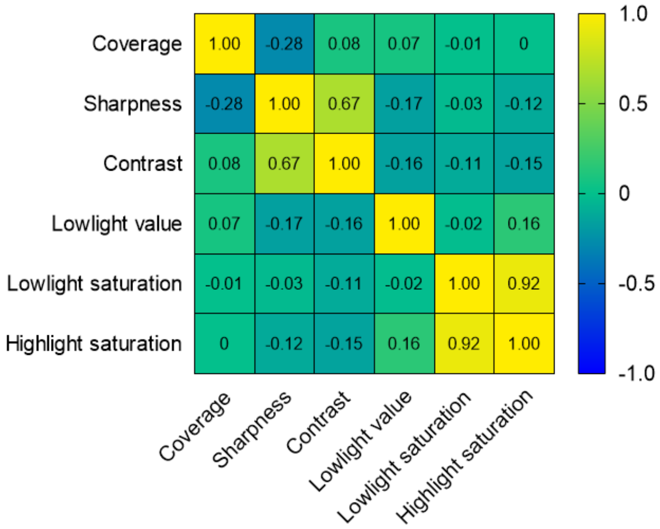


**Supplementary Figure 13.** Heat plot showing Pearson correlations between measured visual features across all stimuli.

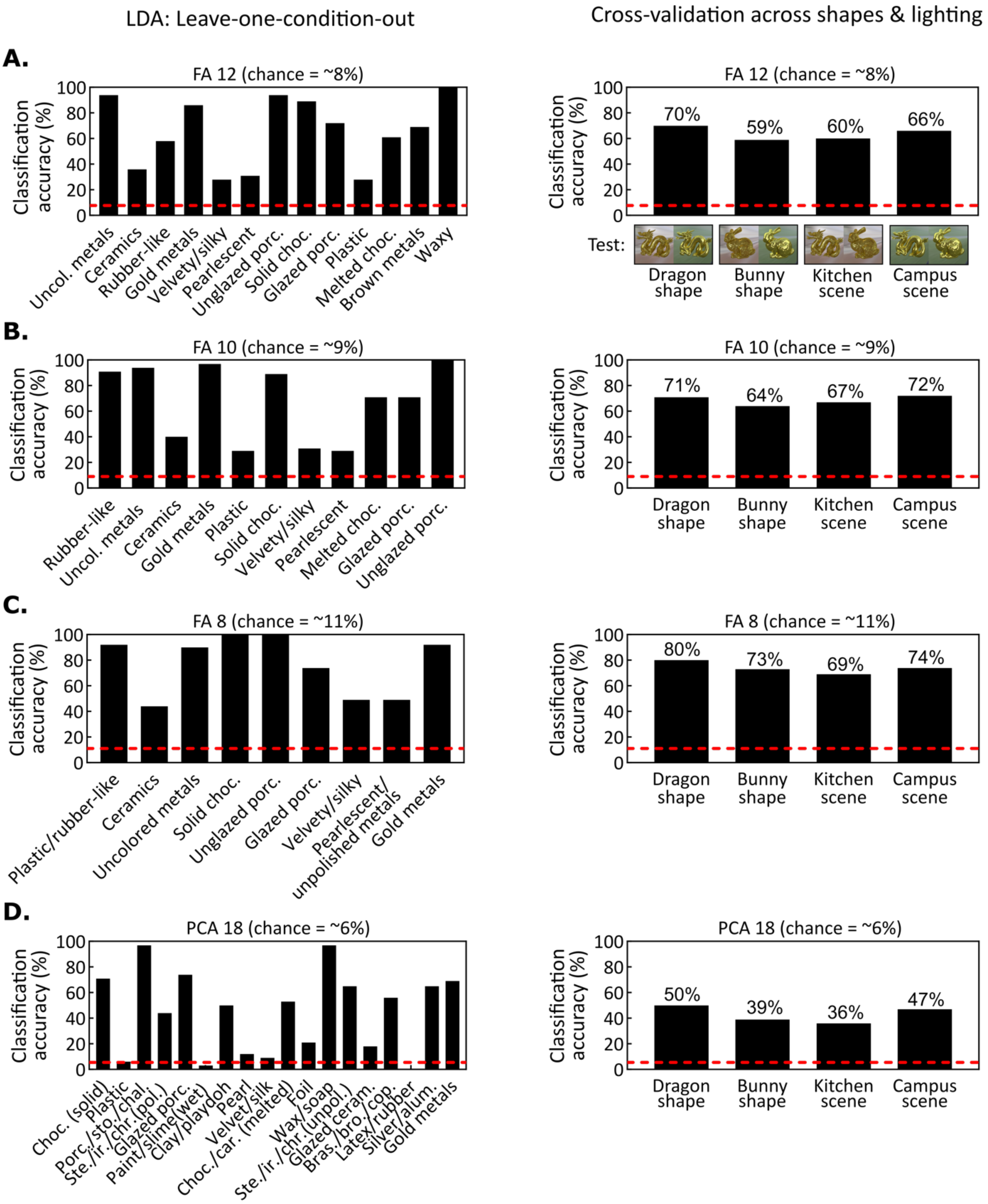


**Supplementary Figure 14.** Results of the linear discriminant analysis with leave one out validation for different factor solutions. Overall, we see the similar classification performance as in the 12-factor solution shown in Figure 6B and C (also shown here in panel A).

| | LD1 | LD2 | LD3 | LD4 | LD5 | LD6 |
|---|---|---|---|---|---|---|
| Coverage | 0.014 | -0.132 | -0.120 | 0.452 | -0.637 | 1.064 |
| Sharpness | -0.106 | 0.199 | 0.016 | 1.533 | -1.049 | 0.051 |
| Contrast | -1.836 | 1.428 | 0.269 | -0.903 | 0.952 | 0.031 |
| Highlight satu. | -0.628 | 1.985 | -2.957 | -0.700 | -1.618 | -0.367 |
| Lowlight satu. | -0.755 | -2.674 | 1.969 | 0.925 | 2.147 | 0.382 |
| Lowlight val. | 1.090 | 1.038 | 0.166 | 0.736 | 0.870 | 0.178 |

**Supplementary Table 1.** Weights of the Linear Discriminant Analysis (LDA) solution using z-scored data. Each column shows the weights on each Linear Discriminant (LD) for each visual feature (rows).

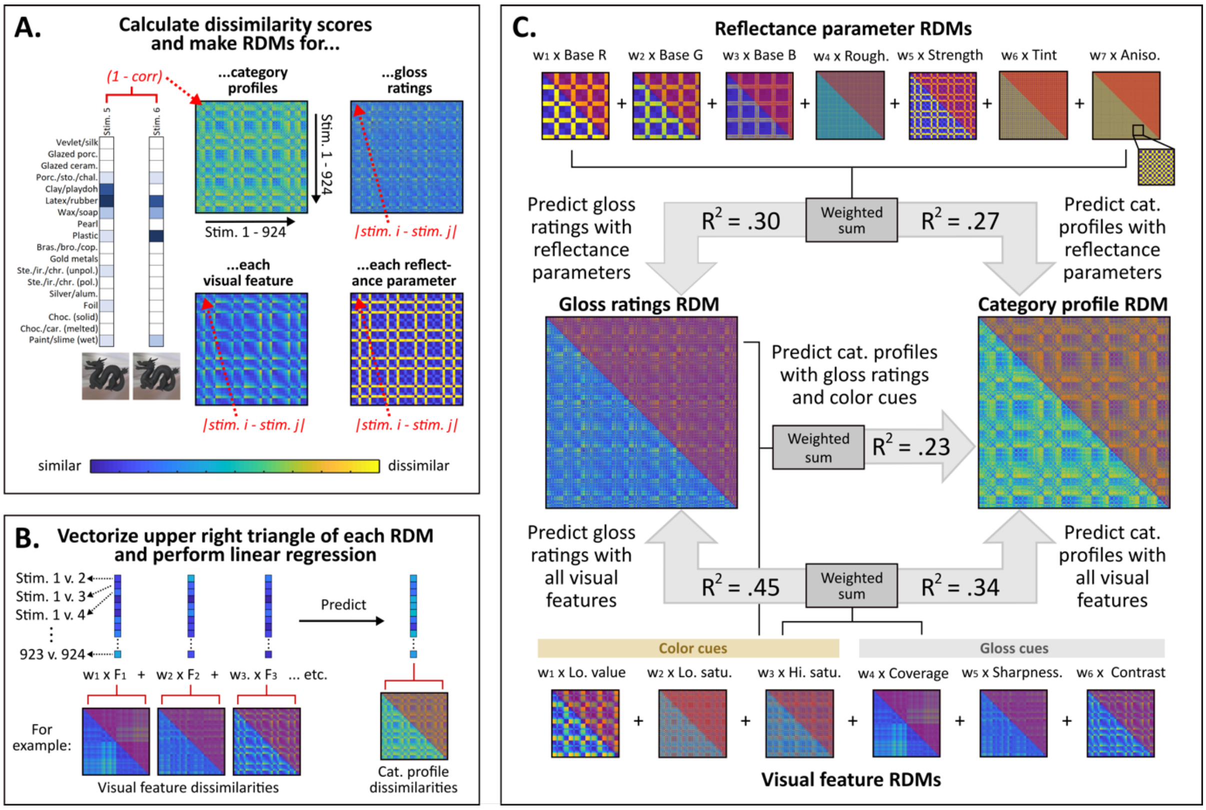


**Supplementary Figure 15.** Representational similarity analysis (RSA) predicts dissimilarities in material and gloss from visual feature differences. **A.** Representational dissimilarity matrix (RDM) for category profiles. Each point in the matrix shows the dissimilarity between two stimuli, calculated as one minus the Spearman correlation (*r*) of their category profiles. We also computed RDMs for average gloss ratings, each of the six visual features, and each reflectance parameter (7), **B.** Dissimilarity scores in the upper triangles of each RDM (highlighted in red) were vectorized and subjected to linear regressions that predicted material dissimilarity and gloss dissimilarity (separately) from feature dissimilarities (panel C, bottom) and reflectance parameter dissimilarities (panel C, top). All RDMs were normalised between zero (most similar) and one (most dissimilar). **C.** The results of each regression analysis. Models based on visual feature dissimilarity outperformed those based on reflectance parameter dissimilarity for both gloss and material.

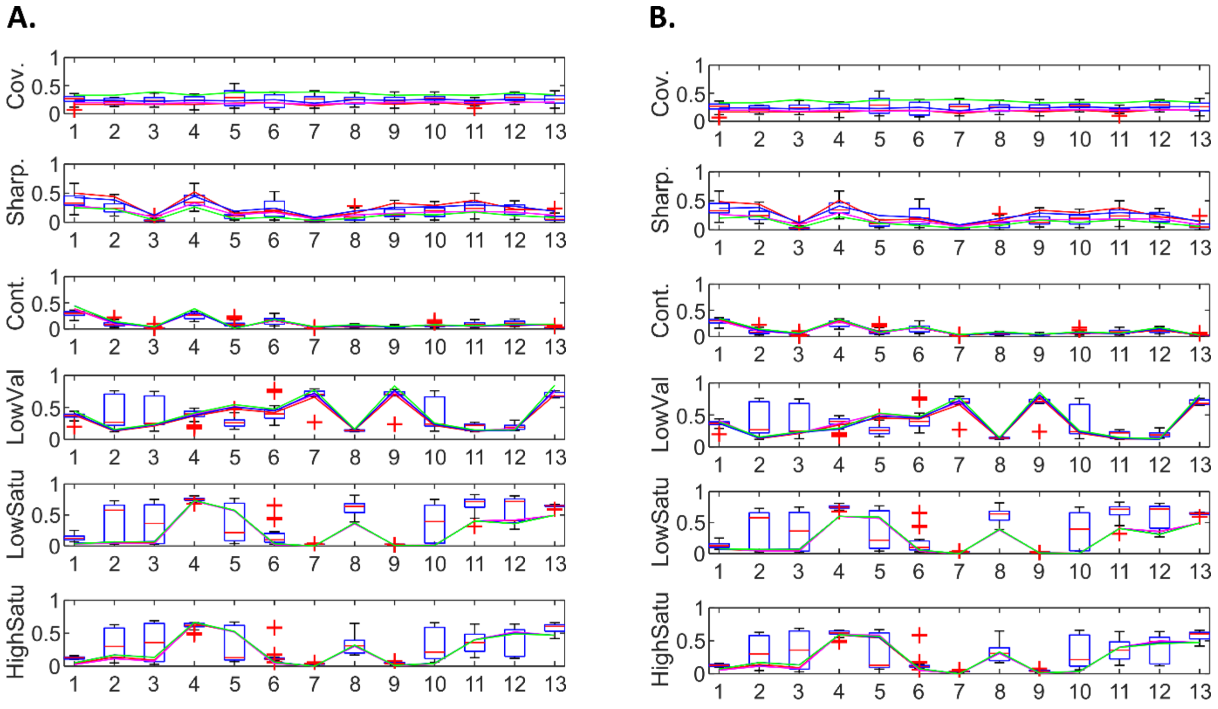


**Supplementary Figure 16.** In order to test whether the visual features we identified were diagnostic for material category, we generated a new stimulus set in Experient 4 through visual feature manipulation (Figure 7 in the manuscript). (A) shows results for the “simple” manipulation condition, with all Gamma (i.e., non-linear) parameters set to 1 and the special velvet filter discarded. (B) shows the manipulation results of the “complex” manipulation condition, involving additional manipulations of the sGamma, dGamma, sBoost and dBoost parameters. The starting point was a rendered image categorized as ‘glazed ceramic’ (Base Color = 0.1, Specular level = 0.3, Specular Tint = 0, Roughness = 0, and Anisotropic level = 0; see Figure 7A). With an approach similar to compositing techniques (Birn, 2014), we generated all remaining material categories by manipulating visual features (see Supplementary Tables 2 and 3), following the steps outlined in the methods section. Values on the x-axes correspond to the emergent material category obtained from factor analysis on the stimulus profiles (Figure 3B). The categories are in same order as in Supplementary Figure 8. For each visual feature we show how the values for this feature were distributed for the original stimuli in this material category (box plots, n=36 stimuli per plot, with the center, minima and maxima denoting the median, lower and upper quartile respectively, whiskers placed at 1.5 times the inter-quarter range, and ‘+’ symbols representing outliers, same data as in Figure 6A), as well as the feature values obtained through our manipulation (colored lines). Here, the different colors represent the 4 shape-light probe combinations in our study (red: Dragon/Kitchen, magenta: Bunny/Kitchen, blue: Dragon/Campus, green: Bunny/Campus). Two observations can be made: (1) visual feature values obtained through feature manipulation all pass through the box plots, suggesting that our manipulation indeed resulted in the appropriate feature values for each category; (2) overall, feature values are quite similar across shape-light probe combinations, yet some features vary slightly more across shape-light probe combinations, e.g., sharpness and coverage which are more affected by different lighting and geometry. We also applied the velvet filter in this condition (only for the velvet manipulation).

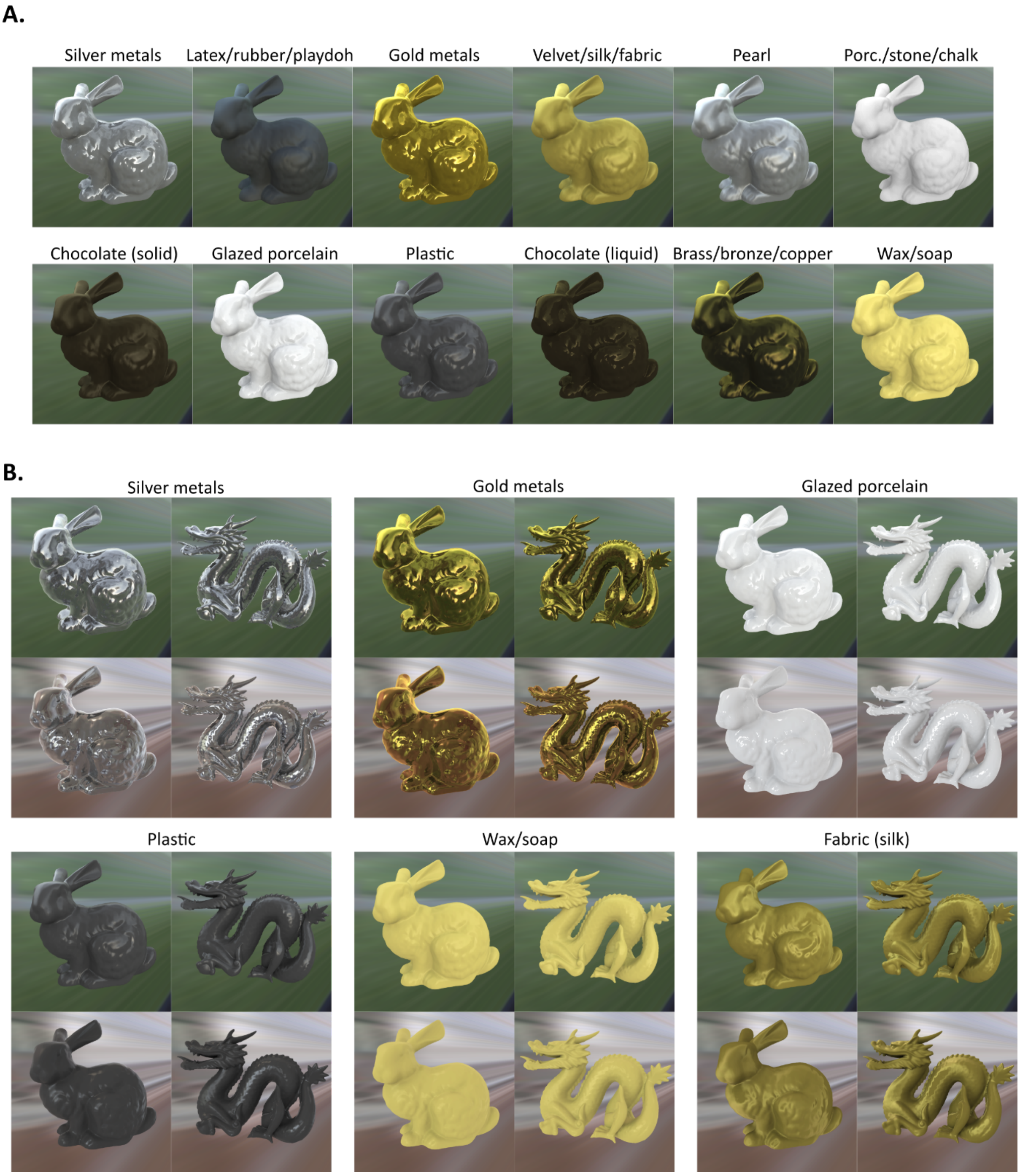


**Supplementary Figure 17.** Example stimuli from the feature manipulation experiment (Experiment 4), labelled with the intended categories. **A.** Examples from the simple feature manipulations on a single scene (bunny object, campus lighting). **B.** Examples of the effects of adding the complex feature manipulations for all four scenes. Using gamma remapping permits to raise the intensity of lowlight reflections in silver and gold manipulations. A similar gamma remapping permits to soften the shadows of the diffuse component in the glazed porcelain, plastic, and wax manipulations, hence mimicking diffusion inside the material. The specific filter for silk fabric creates pairs of elongated highlights with sharp boundaries, which mimics the effect of anisotropy found in such materials.

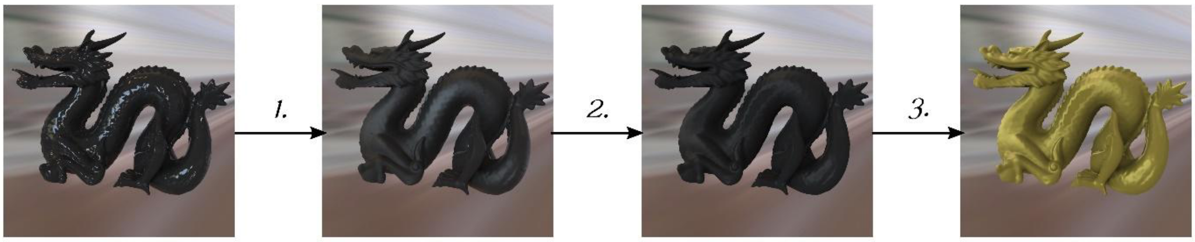


**Supplementary Figure 18.** Image manipulation process used to mimic a satin/velvet appearance (Experiment 4). 1. The specular component of the input image ("Glazed ceramic") is blurred using a large kernel. 2. A non-linear intensity remapping is applied to the specular term only; it has the effect of darkening the centre of highlights, creating elongated highlights with sharper borders. 3. The colour and intensity of the diffuse and specular components are adjusted as with other material manipulations.

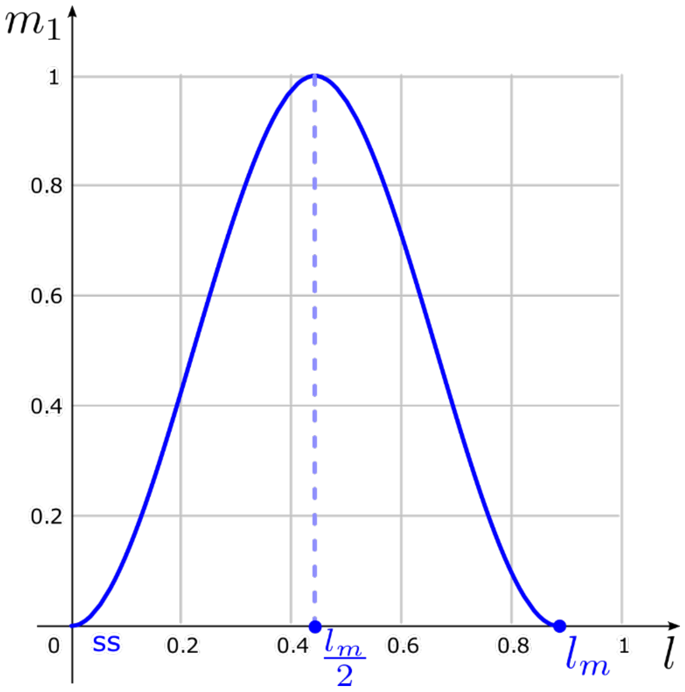


**Supplementary Figure 19.** The non-linear intensity remapping that was applied to the specular term during feature manipulations (Experiment 4).

| Category | Blur | sCol | sSat | sBoost | sGamma | dCol | dSat | dBoost | dGamma |
|---|---|---|---|---|---|---|---|---|---|
| Glazed ceramic | 0 | (1,1,1) | 1 | 1 | 1 | (1,1,1) | 1 | 1 | 1 |
| Brass/Bronze/Copper | 0.45 | (1,0.78,0) | 0.6 | 1.8 | 1 | (1,0.87,0) | 0.25 | 0.75 | 1 |
| Porc./Stone/Chalk | 1 | (1,1,1) | 0 | 0.4 | 1 | (1,1,1) | 0 | 5.5 | 1 |
| Chocolate (liquid) | 0.05 | (1,0.84,0) | 0.4 | 0.5 | 1 | (1,0.75,0) | 0.4 | 1.4 | 1 |
| Chocolate (solid) | 0.5 | (1,0.84,0) | 0.2 | 0.75 | 1 | (1,0.75,0) | 0.4 | 1.4 | 1 |
| Glazed Porcelain | 0 | (1,1,1) | 0 | 0.25 | 1 | (1,1,1) | 0.3 | 6 | 1 |
| Gold metals | 0.15 | (1,0.74,0) | 0.6 | 2.25 | 1 | (0,0,0) | 0.75 | 2.75 | 1 |
| Pearl | 0.525 | (1,1,1) | 0.5 | 1.625 | 1 | (1,1,1) | 0.7 | 3 | 1 |
| Plastic | 0.35 | (1,1,1) | 0.625 | 1 | 1 | (1,1,1) | 0 | 1.75 | 1 |
| Latex/Rubber/Playdoh | 0.75 | (1,1,1) | 1.25 | 0.75 | 1 | (1,1,1) | 1 | 1.5 | 1 |
| Silver metals | 0.125 | (1,1,1) | 0.25 | 1.25 | 1 | (0,0,0) | 1 | 3 | 1 |
| Wax/Soap | 0.35 | (1,1,1) | 0 | 0.25 | 1 | (1,0.84,0) | 0.5 | 6.25 | 1 |
| Velvet/Silk/Fabric | 0.6 | (1,0.95,0) | 0.33 | 1.25 | 1 | (1,0.82,0) | 0.6 | 3.75 | 1 |

**Supplementary Table 2.** Image manipulation parameters for the simple feature manipulations. Note that for glazed ceramic, all parameters have the default value since we start from this category for manipulations. Moreover, the gamma parameters for the specular (sGamma) and diffuse (dGamma) components remain at the default value of 1 for the simple feature manipulations. Note that only the green channel of the specular and diffuse are modified. Indeed, we systematically chose bright and saturated colours and relied on intensity and saturation adjustments to obtain more desaturated colours.

| **Category** | **Blur** | **sCol** | **sSat** | **sBoost** | **sGamma** | **dCol** | **dSat** | **dBoost** | **dGamma** |
|---|---|---|---|---|---|---|---|---|---|
| Glazed ceramic | 0 | (1,1,1) | 1 | 1 | 1 | (1,1,1) | 1 | 1 | 1 |
| Brass/Bronze/Copper | 0.45 | (1,0.78,0) | 0.6 | **3** | **1.4** | (1,0.87,0) | 0.25 | **1.25** | **1.2** |
| Porc./Stone/Chalk | 1 | (1,1,1) | 0 | 0.4 | 1 | (1,1,1) | 0 | 5.5 | 1 |
| Chocolate (liquid) | 0.05 | (1,0.84,0) | 0.4 | 0.5 | 1 | (1,0.75,0) | 0.4 | 1.4 | 1 |
| Chocolate (solid) | 0.5 | (1,0.84,0) | 0.2 | **1.6** | **1.5** | (1,0.75,0) | 0.4 | 1.4 | 1 |
| Glazed Porcelain | 0 | (1,1,1) | 0 | **1** | **1.8** | (1,1,1) | 0.3 | **2.3** | **0.5** |
| Gold metals | 0.15 | (1,0.74,0) | 0.6 | **1.625** | **0.42** | (0,0,0) | 0.75 | 0 | 1 |
| Pearl | 0.525 | (1,1,1) | 0.5 | **1.1** | **0.6** | (1,1,1) | 0.7 | 2.37 | 1 |
| Plastic | 0.35 | (1,1,1) | 0.5 | **0.8** | **1.3** | (1,1,1) | 0 | **1.5** | **0.9** |
| Latex/Rubber/Playdoh | 0.75 | (1,1,1) | 1.25 | 0.75 | 1 | (1,1,1) | 1 | 1.5 | 1 |
| Silver metals | 0.125 | (1,1,1) | 0.25 | **1.25** | **0.3** | (0,0,0) | 1 | 0 | 1 |
| Wax/Soap | 0.35 | (1,1,1) | 0 | **0.13** | **0.7** | (1,0.84,0) | 0.5 | **2.2** | **0.5** |
| Velvet/Silk/Fabric | 0.6 | (1,0.95,0) | 0.33 | 2.9 | 1 | (1,0.82,0) | 0.6 | 3.75 | 1 |

**Supplementary Table 3.** Filter parameters for the “complex” feature manipulation condition. Modified parameters compared to Supplementary Table 2 are shown in bold.

## SUPPLEMENTAL REFERENCES


Birn, J. (2014). Digital Lighting and Rendering. Chapter 11: In K. Johnson (Ed.). *Rendering in layers and passes for compositing* (third edition, 361-409). IN, USA: New Riders Pub.

Hassen, R., Wang, Z., & Salama, M. M. A. (2013). Image sharpness assessment based on local phase coherence. *IEEE Transactions on Image Processing, 22*(7), 2798-2810. (https://ece.uwaterloo.ca/~z70wang/research/lpcsi/)

Norman, J. F., Todd, J. T. & Phillips, F. (2020). Effects of illumination on the categorization of shiny materials. *Journal of Vision, 20(5)*, 1-16.
