# Supplementary material for: Material category of visual objects computed from specular image structure
Source: Nat Hum Behav. 2023 Jun 29;7(7):1152–69. doi: 10.1038/s41562-023-01601-0 (PMC10365995; doi:10.1038/s41562-023-01601-0)
Supplement: Supplementary file 2 — Reporting Summary [file 41562_2023_1601_MOESM2_ESM.pdf]

nature research

Corresponding author(s): Alexandra C. Schmid

Last updated by author(s): Mar 29, 2023

Reporting Summary

Nature Research wishes to improve the reproducibility of the work that we publish. This form provides structure for consistency and transparency in reporting. For further information on Nature Research policies, see Authors & Referees and the Editorial Policy Checklist.

Statistics

For all statistical analyses, confirm that the following items are present in the figure legend, table legend, main text, or Methods section.

n/a	Confirmed	
☐	☒	The exact sample size (n) for each experimental group/condition, given as a discrete number and unit of measurement
☐	☒	A statement on whether measurements were taken from distinct samples or whether the same sample was measured repeatedly
☒	☐	The statistical test(s) used AND whether they are one- or two-sided<br>*Only common tests should be described solely by name; describe more complex techniques in the Methods section.*
☒	☐	A description of all covariates tested
☐	☒	A description of any assumptions or corrections, such as tests of normality and adjustment for multiple comparisons
☐	☒	A full description of the statistical parameters including central tendency (e.g. means) or other basic estimates (e.g. regression coefficient) AND variation (e.g. standard deviation) or associated estimates of uncertainty (e.g. confidence intervals)
☐	☒	For null hypothesis testing, the test statistic (e.g. F, t, r) with confidence intervals, effect sizes, degrees of freedom and P value noted<br>*Give P values as exact values whenever suitable.*
☒	☐	For Bayesian analysis, information on the choice of priors and Markov chain Monte Carlo settings
☒	☐	For hierarchical and complex designs, identification of the appropriate level for tests and full reporting of outcomes
☐	☒	Estimates of effect sizes (e.g. Cohen's d, Pearson's r), indicating how they were calculated

Our web collection on statistics for biologists contains articles on many of the points above.

Software and code

Policy information about availability of computer code

Data collection	Stimuli were rendered using the open-source modelling software Blender (v2.79). Stimulus presentation and data collection were controlled by a MATLAB script (release 2018b, Mathworks, Natick, MA) using the Psychophysics Toolbox (v3; Brainard, 1997).
Data analysis	Data analysis was performed using MATLAB scripts (release 2018b, Mathworks, Natick, MA). Code for analyses (including image analyses) are available on Zendo: DOI: 10.5281/zenodo.5080227.

For manuscripts utilizing custom algorithms or software that are central to the research but not yet described in published literature, software must be made available to editors/reviewers. We strongly encourage code deposition in a community repository (e.g. GitHub). See the Nature Research guidelines for submitting code & software for further information.

Data

Policy information about availability of data

All manuscripts must include a data availability statement. This statement should provide the following information, where applicable:

- Accession codes, unique identifiers, or web links for publicly available datasets
- A list of figures that have associated raw data
- A description of any restrictions on data availability

Psychophysics data and stimuli used in the experiments are available on Zendo: DOI: 10.5281/zenodo.5080227. The 3D meshes of the bunny and dragon objects were obtained from the Stanford 3D Scanning Repository and can be found under the following link: http://graphics.stanford.edu/data/3Dscanrep/.

Field-specific reporting

Please select the one below that is the best fit for your research. If you are not sure, read the appropriate sections before making your selection.

☐ Life sciences ☒ Behavioural & social sciences ☐ Ecological, evolutionary & environmental sciences

For a reference copy of the document with all sections, see nature.com/documents/nr-reporting-summary-flat.pdf

Behavioural & social sciences study design

All studies must disclose on these points even when the disclosure is negative.

Study description	The study had a within-subject experimental design, with quantitative data for all experiments.
Research sample	Participants were undergraduate students from the psychology programme at Justus Liebig University Giessen in Germany. Fifteen participants completed the free-naming experiment (Experiment 1, mean age: 23.7, female 80%, male 20%). Eighty native-level German speakers participated in the 18-AFC experiment (Experiment 2, mean age 24.9, female: 83.3%, male: 16.7%), 22 participants took part in the gloss rating experiment (Experiment 3, mean age: 25.3, female: 58.3%, male: 41.7%), and 22 participants took part in the feature manipulation experiment (Experiment 4, mean age: 23.4 , female: 81.8%, male: 18.2%). Different participants were recruited for each experiment.
Sampling strategy	The experiment was advertised to students at Justus Liebig Univerity through the university's experimental volunteer system and participants were chosen on a first come first served basis. Sample size was chosen based on standards in the field, i.e., psychophysical studies of mid-level perception, and was slightly higher than this standard (e.g., 20 participants per experiment in Storrs, Anderson, & Fleming, 2021; Nat. Hum. Behav.).
Data collection	In the free naming task (Experiment 1), stimuli were projected onto a white wall in a classroom. Thick black cloth was used to block light from the windows, so that the only source of light came from the projected image. For all other experiments the stimuli were presented on a Sony OLED monitor running at a refresh rate of 120 Hz with a resolution of 1920 x 1080 pixels controlled by a Dell computer running Windows 10. Stimuli were viewed in a dark room at a viewing distance of approximately 60cm. The only source of light was the monitor that displayed the stimuli. Participants used mouse and keyboard presses to respond to stimuli presented on the screen. For Experiment 1 participants recorded their responses using pen and paper. Only experimenter and participant were present during the experiment.<br><br>In Experiment 1, two researchers were present in the same room as participants. In Experiments 2-4, a researcher was present in the same room as the participant. In all Experiments, researchers were not blinded to the study hypothesis, however our experiments did not involve assignment to groups (but within subject design).
Timing	Experiment 1: 3rd May 2018 (one day only); Experiment 2: 11th March - 14th May 2019; Experiment 3: 14th-20th August 2019; Experiment 4: 11th-21st November 2019
Data exclusions	One participant was excluded from Experiment 3 because they did not understand the task instructions. Not understanding the task was a pre-established reason for excluding participants from analysis.
Non-participation	No participants dropped out/declinded participation.
Randomization	Participants were not allocated into experimental groups (within subjects design).

Reporting for specific materials, systems and methods

We require information from authors about some types of materials, experimental systems and methods used in many studies. Here, indicate whether each material, system or method listed is relevant to your study. If you are not sure if a list item applies to your research, read the appropriate section before selecting a response.

Materials & experimental systems

n/a	Involved in the study	
☒	☐	Antibodies
☒	☐	Eukaryotic cell lines
☒	☐	Palaeontology
☒	☐	Animals and other organisms
☐	☒	Human research participants
☒	☐	Clinical data

Methods

n/a	Involved in the study	
☒	☐	ChIP-seq
☒	☐	Flow cytometry
☒	☐	MRI-based neuroimaging

Human research participants

Policy information about studies involving human research participants

Population characteristics	See above.
Recruitment	Participants volunteered via a university experimental volunteer system, and participants were chosen on a first come first served basis. Participants were chosen based on their availability at the time of data collection. Visual perception of materials likely varies somewhat with environmental and cultural exposure to different materials. We consider our sample of educated young adults to be representative of visual material perception within industrialised Western countries, but not necessarily representative of all humans.<br><br>Any potential self selection biases such as financial pressure, or motivation to gain experience in a psychological experience are not likely to impact the generalisability of the results, since our investigation focuses on basic perceptual mechanisms.
Ethics oversight	This study was approved by the local ethics review board of the Justus Liebig University Giessen (LEK FB 06) and strictly adhered to the ethical guidelines put forward by the declaration of Helsinki (2013). All participants gave written informed consent prior to the experiments and were told about the purpose of the experiments. All participants were compensated for their participation at a rate of 8 €/hour.

Note that full information on the approval of the study protocol must also be provided in the manuscript.
